# Progress in Medicine

**Published:** 1896-03-14

**Authors:** 


### Progress in Medicine.

### DISEASES OF THE NERVOUS SYSTEM.

(*Continued from page* 382.)

**Insanity.—Pathology.**—Writing on the psychological epochs that predispose to insanity, with observations on the management of each, W. P. Stirling[8] remarks that there are certain well-defined physiological epochs that appear at fixed periods during the growth and development of the body. These epochs are all strictly physiological in the order and nature of their occurrence, save one, the epoch of heredity, and that may be said to be pathological, or patho-psychological. These epochs are six in nmber, and he designates them as follows: (1) Early childhood, (2) puberty, (3) maternity, (4) heredity, (5) menopause, (6) senility. The epoch of early childhood covers the period from the sixth month to the seventh year. Strictly speaking, the period should be regarded as an indirect epoch only, since insanity occurring so early in life is extremely rare. There are, however, frequent cases in which the accidents and incidents of early life facilitate the establishment of insanity in after years. The epoch of puberty in the female extends from the thirteenth to the fifteenth year; in the male, from the fourteenth to the sixteenth year. The third epoch covers the entire child-bearing period from the fifteenth to the forty-fifth year. Insanity occurs more frequently during the first than during the last half of the period. The epoch of heredity is an important and variable one, and embraces that critical period in the life of every individual whose ancestors were accustomed to exhibit evidences of insanity on reaching a certain age. The fifth epoch embraces the climacteric, and extends in women from the forty-third to the forty-fifth year.

As regards the accidents and incidents of the first epoch which render the subject prone to mental disorder, the most important is dentition, and especially in conjunction with rickets. Syphilis, chronic alcoholism, insanity, or epilepsy, in the parent, stamp on the nervous system of the child a congenital taint that unfits it to cope with the serious disturbances that mark the steps of growth and development. Genuine epilepsy probably never occurs in the first instance out of difficult dentition alone, though epileptiform convulsions may, and these in time may eventuate in all the characteristics of genuine epilepsy. That insanity often follows in the footsteps of epilepsy is a matter of common knowledge. Besides dentition, there are a great variety of other causes, *e.g.*, traumatism, infantile paralysis, effects of heat, mental shock, fevers of specific origin, diphtheria, whooping-cough, strictures, and malformations of the genital organs.

The author draws attention to the difference in the insanity of puberty in the male and female. The male attains maturity gradually, whereas the female acquires it almost at a bound. The insanity of the male at this period is characterised by a silly, childish disposition, depression and melancholia, and mental enfeeblement. In the female, insanity is at this time essentially an acute neurosis, and acute mania is the form of insanity that is most frequent.

As regards the other periods of insanity, we may quote the author's directions as to treatment. In acute mania, following childbirth, the treatment is most important; in the majority of cases it is a good practice to begin with a saline aperient. We should search for the cause of the fever; it may be due to putrescible matter in the uterine cavity, or to septic absorption through a raw surface, or due to peritonitis, phlebitis, or an inflamed mammary gland, and these conditions require appropriate treatment. If the vitiated condition of the blood demands the free use of iron, the author warns against the giving of any chalybeate preparations until all traces of acute excitement have subsided. The infant must be early removed from the mother's care. The diet should consist of milk, beef tea, broth, and eggs, in small quantities frequently. For the insomnia, potassium bromide, sulfonal, or paraldehyde are commended, preferably the latter, in two-drachm doses, in a small quantity of port wine. Warm baths, however, are often effectual, and should be tried before drugs are given. For the melancholia of the climacteric, a tonic regimen, outdoor life, strict attention to dietetic matters, and change of environment are indicated, and for anæmia iron preparations. Sleep generally requires to be induced by drugs.

In the insanity of old age opium is generally most beneficial, especially when there is great restlessness and fixed insomnia.

**Relation of Insanity to Phthisis.**—H. A. Tomlinson,[9] superintendent of the St. Peter State Hospital, has reached the conclusion that the concurrence and coexistence of phthisis and insanity indicate an imperfectly developed or unstable organism with a materially lessened power of resistance, and that this hereditary or acquired instability or vulnerability manifests itself in the lungs of one individual, making him, under proper environment, liable to the development of phthisis; while in another, the instability being in the nervous system, the tendency would be to develop insanity. He goes on to consider what constitutes this vulnerability, and why there should be such an intimate association between these two conditions, which are not necessarily concomitants, and which, as a rule, exist independently. However, the offspring of those individuals who are subjects of the tuberculous tendency are very likely to be insane; indeed, the writer states that in his experience phthisis and insanity are equally potent factors in the production of brain instability. Tomlinson found that out of 695 patients admitted between August, 1892 and 1895, 70 had a history of phthisis in the family. He gives several interesting analytical tables.

An instructive analysis of 156 admissions to the St. Lawrence State Hospital is given by Mosher,[10] of which we can only give his summary :—

| | Delirium. | Stupor. | Melancholia. | Mania. | Subacute Forms. | Totals. |
|---|---|---|---|---|---|---|
| Cases ... ... ... ... ... ... | 9 | 5 | 23 | 16 | 6 | 59 |
| Completed cases ... ... ... ... ... | 7 | 5 | 16 | 6 | 3 | 37 |
| Recoveries ... ... ... ... ... ... | 5 | 3 | 10 | 5 | 2 | 25 |
| Per cent. recoveries on completed cases ... | 71½ | 60 | 62½ | 83⅓ | 66⅔ | 67½ |
| Deaths ... ... ... ... ... ... | 0 | 2 | 3 | 1 | 0 | 6 |
| Per cent. deaths... ... ... ... ... | — | 40 | 14 | 6 | — | 10 |
| Average gain in weight of recovered cases ... | 11 lbs. | 17 lbs. | 16½ lbs. | 24 lbs. | 6½ lbs. | 15 lbs. |
| Duration before admission ... ... ... | 9 days. | 2 months. | 5½ months. | 6 weeks. | 8½ months. | 3⅗ months. |
| Period under treatment of recovered cases... | 14 weeks. | 3 months. | 4 months. | 5 months. | 14 weeks. | 4 months. |

In a study of hysteria and hypochondriasis, C. E. Lockwood,[11] having enumerated conditions necessary to promote healthy brain action, gives the following as causes productive of unhealthy brain action : (1) Hereditary transmission ; (2) improper mental and physical training, with unsuitable environment; (3) physical defects interfering with general healthy nutrition; (4) lack of adoption of definite principles and formation of fixed habits which tend to the making of a well-balanced mind; (5) constant indulgence of animal appetite against the voice of conviction, involving weakening of self-control; (6) lack of motive in life, occupation, &c., allowing too much introspection; (7) harbouring of beliefs based on superstition, involving the stifling of reason; (8) the excessive use of alcoholic stimulants, opium, and tobacco; (9) lack of proper mental diversion and physical exercise; (10) low state of health, especially in connection with sexual organs; (11) sudden climatic alterations; (12) toxic matters in the blood, leading to unhealthy nutrition; (13) the tendency of the thought and practice of the present day among young men and women, which seems to be antagonistic to the consummation of marriage, and the assumption of the ties and interests of motherhood and fatherhood; (14) the influence of a degenerate literature and art, inculcating the following of the lead of the emotions, and the consequent stimulation of the lower centres and lowering of the influence of reason and self-control.

The writer states that there are two affections to which humanity is liable—hysteria and hypochondriasis—which, in his opinion, are peculiarly the result of defective action of the higher centres, the reason and the will, in their inhibitory action on the lower centres. Hysteria is a yielding of the mind to any morbid impulse that enters it; hypochrondriasis is a mind habit of dwelling on bodily functions until it becomes a second nature, and the mind becomes in this way sensible to impressions which, in health, are unperceived. The causes of the conditions are physical and mental, and both demand careful attention for successful treatment.

The home treatment of insanity is a question of the first importance, and as the natural inclination of friends is to prevent the removal of a patient to an asylum as long as it can be avoided, often too long for the patient's ultimate welfare, it is a question that demands the careful consideration of experts and a definite answer as to its advisability. H. M. Bannister[12] discusses the subject under two heads: (1) Can insanity be as well treated outside of an asylum as within its walls? (2) Has the asylum any direct curative influence in cases of insanity? Confining the elastic term "insanity" to acute or curable cases we may understand by it ordinary cases of mania and melancholia, which form almost the whole of "curable cases," with the exception of a few cases of toxic delirium and primary dementia. Apart from the question of expense, which must decide in favour of asylum treatment in a large proportion, the author calls attention to the great difficulty involved in mechanical restraint, which in private houses is necessary, where nothing of the same kind is demanded in an asylum. There is also a difficulty in obtaining trained attendants for casual cases, while there is also the disadvantage to the insane patient in retaining him in accustomed surroundings, which add to the irritation of any necessary but unaccustomed restraint. The author does not consider there is so much difficulty attending the depressive forms of insanity at home, provided there is no suicidal tendency, often a difficult matter to determine. The only forms of acute insanity that can advisably be treated at ordinary homes are certain types of primary dementia and certain delirious forms of exhausting disease, with great physical prostration. On the other hand, the author urges the well-recognised therapeutic influence of a well-regulated discipline that an asylum should display, while the mere removal from domestic irritations and responsibilities has in itself often a most beneficial effect.

[8] Med. Rec., Oct. 19, 1895. [9] Journ. of Nerv. and Ment. Dis., Vol. XX., No. 10. [10] Med. Rec., Dec. 14, 1895. [11] Med. Rec., Nov. 23, 1896. [12] Journ. of Nerv. and Mental Dis., Nov., 1895.

